# Progression and Pathology of Traumatic Optic Neuropathy From Repeated Primary Blast Exposure

**DOI:** 10.3389/fnins.2019.00719

**Published:** 2019-07-11

**Authors:** Alexandra Bernardo-Colón, Victoria Vest, Melissa L. Cooper, Sarah A. Naguib, David J. Calkins, Tonia S. Rex

**Affiliations:** ^1^Vanderbilt Eye Institute, Vanderbilt University Medical Center, Nashville, TN, United States; ^2^Department of Ophthalmology and Visual Sciences, Vanderbilt University School of Medicine, Nashville, TN, United States

**Keywords:** optic neuropathy, axon degeneration, axonopathy, blast, indirect traumatic optic neuropathy, repeat neurotrauma, intraocular pressure, retinal ganglion cells

## Abstract

Indirect traumatic optic neuropathy (ITON) is a condition that is often associated with traumatic brain injury and can result in significant vision loss due to degeneration of retinal ganglion cell (RGC) axons at the time of injury or within the ensuing weeks. We used a mouse model of eye-directed air-blast exposure to characterize the histopathology of blast-induced ITON. This injury caused a transient elevation of intraocular pressure with subsequent RGC death and axon degeneration that was similar throughout the length of the optic nerve (ON). Deficits in active anterograde axon transport to the superior colliculus accompanied axon degeneration and first appeared in peripheral representations of the retina. Glial area in the ON increased early after injury and involved a later period of additional expansion. The increase in area involved a transient change in astrocyte organization independent of axon degeneration. While levels of many cytokines and chemokines did not change, IL-1α and IL-1β increased in both the ON and retina. In contrast, glaucoma shows distal to proximal axon degeneration with astrocyte remodeling and increases in many cytokines and chemokines. Further, direct traumatic optic neuropathies have a clear site of injury with rapid, progressive axon degeneration and cell death. These data show that blast-induced ITON is a distinct neuropathology from other optic neuropathies.

## Introduction

Indirect traumatic optic neuropathy (ITON) is a condition in which the optic nerve (ON) degenerates in the absence of a penetrating injury. It is a rare condition in the general U.S. population but occurs in 0.5–5.0% of patients with a traumatic brain injury (TBI) (Steinsapir and Goldberg, 1994; Sarkies, 2004). Approximately 50% of ITON patients present with immediate, severe vision loss even though the optic disk appears normal (Yu-Wai-Man, 2015). Four to six weeks after injury the optic disk typically develops pallor, indicative of atrophy (Agrawal et al., 2013). During this time approximately 10% of ITON patients exhibit further vision loss (Yu-Wai-Man, 2015). Interestingly, another subset of patients show some improvement over this time. The rarity and complexity of this condition has made clinical studies and trials challenging. Currently there are no therapies that have been shown to be more effective than observation alone.

We have developed a model of ITON in which an air-blast is directed at the eye to avoid causing a TBI and associated cortical visual system damage that could confound results (Hines-Beard et al., 2012; Bernardo-Colon et al., 2018). Repeated low level air blast exposure with our system damages the ON while avoiding damage to the photoreceptors unlike a single, greater magnitude blast or blunt force injury (Bernardo-Colon et al., 2018; Vest et al., 2019). This repeat injury paradigm is particularly relevant to the military population due to exposure to linked exposives (mines), large firearms, or improvised explosive devices, which result in multiple blast exposures in combination with blunt force injuries in a very short time scale. Our repeat blast paradigm induces 32% axon degeneration in the ON at 2 weeks after injury. The goal of this study was to examine the pathophysiology of axon degeneration after blast-induced ITON in order to better inform the development of treatment strategies. We assessed the intraocular pressure (IOP), progression of retinal ganglion cell (RGC) loss, axon degeneration, active anterograde transport capacity, and ON glial morphology and responses including levels of cytokines and chemokines.

## Materials and Methods

### Mice

Three month old male C57Bl/6 mice were purchased from Jackson Laboratories (Bar Harbor, ME, United States). We previously detected no differences between male and female mice and most service members are male. All procedures were performed in accordance with AALAC and the Association for Research in Vision and Ophthalmology guidelines and the VUMC Institutional Animal Care and Use Committee approved protocol. Mice were perfused with PBS and 4% paraformaldehyde at collection.

### Trauma Model

Injury was induced as previously described (Bernardo-Colon et al., 2018; Vest et al., 2019). Briefly, mice were anesthetized with 2.5% isofluorane and secured into a padded housing chamber. The housing chamber was placed inside of a PVC pipe. The left eye of the mouse was positioned against the hole in the pipe, which was aligned with the barrel of the paintball marker. All experiments were performed in the morning. Mice were exposed to two 15 psi blasts of air at a 0.5 sec interval per day for 3 days. Sham mice were anesthetized, placed into the housing chamber and exposed to the sound but not the air-blast due to obstruction of the air prior to it reaching the eye. Mice were provided gel recovery food (Clear H_2_O, Portland, ME, United States) for the first 3 days post-injury.

### IOP Measurement

Intraocular pressure was measured using the Icare TonoLab rebound tonometer (Colonial Medical Supply, Franconia, NH, United States). Mice were anesthetized using isoflurane, and 10 measurements were acquired from each eye within 2 min of induction of anesthesia. IOP was measured in mice immediately prior to the first blast exposure and then over time after the last blast exposure.

### Anterograde Transport Tracing

Mice were anesthetized with isoflurane and injected intravitreally in the blast-exposed eye with 2 μL cholera toxin B subunit (CTB) conjugated to Alexa Fluor 594 (Thermo Fisher) using a 30 gauge Hamilton syringe. Seventy two hours following injection, mice were anesthetized via intraperitoneal injection of 2,2,2-tribromoethanol (Sigma-Aldrich, Saint Louis, MO, United States) and perfused with 4% paraformaldehyde in PBS. Brains were post-fixed in 4% paraformaldehyde in PBS for 2 days (2d), infiltrated 1 day each in 10, 20, and 30% sucrose for cryo-protection. Brains were then cryo-sectioned coronally at 50 μm thickness. Sections traversing the SC were mounted, and the SC was imaged via epifluorescence microscopy (Nikon Instruments, Melville, NY, United States). Fluorescence in the SC sections was quantified using ImagePro software (Media Cybernetics, Rockville, MD, United States) using an automated macro described earlier to quantify the fraction of the SC retinotopic representation containing intact anterograde transport (Bond et al., 2016). Briefly, fluorescence intensity was summed along the vertical dorsal-ventral columns through the SC sections, and a normalized fluorescence intensity score was generated along the medial-lateral length of the SC. A fluorescence intensity heat map was generated from fluorescence in all SC sections with red representing maximum fluorescence and blue representing no fluorescence. These values were normalized to sham to generate the percent intact transport values. Intact transport is defined as > 70% of maximum fluorescence. We used this data to generate a complete retinotopic map using our previously published protocol (Lambert et al., 2011; Ward et al., 2014). Briefly, the collicular intensity map was overlaid on a retino-collicular projection in Photoshop and manually stretched so that the collicular points directly overlaid the corresponding locations on the retinotopic projection, as established in Drager and Hubel (1976). Areas with transport deficits (less than 70% of maximum fluorescence) were represented with solid gray. The labels around the maps indicate eccentricity coordinates of the visual field. Abbreviations I, N, S, T indicate inferior, nasal, superior, and temporal quadrants of the retina, respectively.

### Immunohistochemistry and Cell Counting

Eyes were preserved in 4% paraformaldehyde, cryoprotected in 30% sucrose overnight at 4°C and embedded in tissue freezing medium (Triangle Biomedical, Durham, NC, United States). Ten-micron thick sections were collected in round on a cryostat (Fisher, Pittsburgh, PA, United States). Each slide had representative sections from the entire eye when collected in round. Slides were then rinsed with PBS and incubated at room temperature in normal donkey serum (NDS; 0.05%) in 0.1M phosphate buffer with 0.5% bovine serum albumin and 0.1% Triton X-100 (phosphate buffer plus Triton X-100 [PBT]) for 2 h. The slides were incubated overnight at 4°C in anti-RBPMS (1:400; ABN1362; Millipore, Burlington, MA, United States) and anti-phosphorylated neurofilament H (SMI 34; 1:1000; 835503; Biolegend, San Diego, CA, United States) in PBT. Slides were then rinsed with PBS and incubated with their appropriate secondary antibody (donkey anti-rabbit 555 and donkey anti-mouse 594; Life Technologies, Carlsbad, CA, United States) for 2 h at room temperature. Finally, slides were rinsed in PBS, mounted in Vectashield Mounting medium with DAPI (Vector Laboratories, Burlingame, CA, United States) or Fluoromount-G (Southern Biotech, Birmingham, AL, United States). Slides were imaged on a Nikon Eclipse epifluorescence microscope (Nikon, Melville, NY, United States). All images were collected from sections through the ON at identical magnification, gain, and exposure settings. These same sections were used for quantification of DAPI-positive cells in the GCL. Nikon software was used to measure the length of the retina that was used for counting.

### ON Histology and Axon Counting

The entire ON, from next to the globe to the optic chiasm, was collected, cut into three equal pieces, post-fixed in glutaraldehyde, and embedded in Resin 812 and Araldite 502 (cat 14900 and 10900, respectively; Electron Microscopy Sciences, Hatfield, PA, United States) according to previously published protocol (Bricker-Anthony et al., 2014, 2017; Bond et al., 2016; Hines-Beard et al., 2016). One-micron thick sections were collected on a Leica EM-UC7 microtome and stained with 1% paraphenylenediamine (ppd) and 1% toluidine blue. Proximal sections were collected from the end of the ON that was cut from the globe. Medial sections were collected from the end of the mid-section of the ON that faced the globe. Distal sections were collected from the distal piece of the ON and from the side that was cut away from the optic chiasm. Cross-sections were imaged on a Nikon Eclipse Ni-E microscope (Nikon Instruments Inc., Melville, NY, United States) using a 100x oil immersion objective. Total and degenerating axons were quantified using Image J. A grid was used to count 20% of the ON cross-sectional surface area to avoid bias.

### Glial Analyses

Glial morphology and organization were analyzed as previously described (Cooper et al., 2016, 2018). Briefly, Matlab functions were used to quantify the fraction of total nerve area covered by glia, parallelism of astrocyte processes, and spatial distribution of astrocyte processes radially across the nerve. Higher parallelism values indicate a greater degree of alignment of the glial processes, while lower values indicate more disorder. Spatial distribution, specifically bias toward the edge or center of the nerve, was quantified by defining a center of mass (CoM) based on division of the nerve into concentric rings. Values near 10 indicate an even distribution; values less than 10 indicate bias toward the edge of the nerve; and values greater than 10 indicate bias toward the center of the nerve. For each nerve, these metrics were plotted against each other, as well as against other disease indices (total axon count, axon density, nerve size, and time post-injury) to determine trends in disease progression. For each index pair, linear regression analysis was performed in SigmaPlot. A *p*-value less than 0.05 was considered to indicate significant correlation.

### Multiplex ELISA

Retinas were sonicated and run in singlet on the Milliplex MAP Mouse Cytokine/Chemokine Magnetic Bead Panel I 25-plex assay per manufacturer’s protocol (EMD Millipore, Billerica, MA, United States). Plates were read on a Luminex MAGPIX with xPONENT software (Thermo Fisher Scientific, Waltham, MA, United States) using settings stated in the manufacturer’s protocol. Multiplex ELISA plates were read and analyzed through the use of the Vanderbilt Hormone Assay and Analytical Services Core.

### Statistical Analysis

Graphpad Prism software was used to perform statistical analysis of all data. A minimum of four ONs was assessed per group for axon quantification. A one-way ANOVA and Tukey’s *post hoc* test was performed. These numbers are based on our previous statistical analysis for these assays (Bond et al., 2016; Hines-Beard et al., 2016).

## Results

### Blast-Exposure Causes a Short-Term Elevation in IOP

**FIGURE 1:**
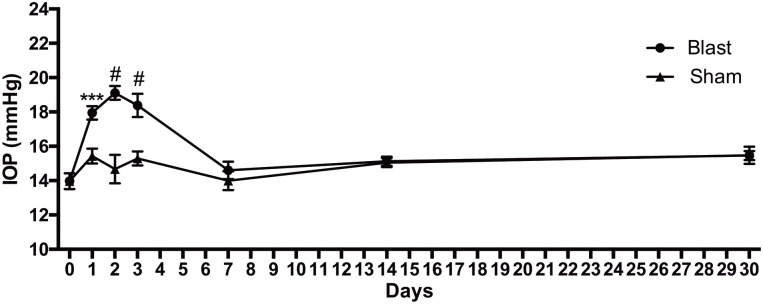
IOP changes over time after blast injury. Student’s *t*-test comparisons show elevations in IOP in the blast-exposed group as compared to shams at 1, 2, and 3 days after blast. ^∗∗∗^*p* < 0.001; #*p* < 0.0001. Baseline, *n* = 14; 1 day sham, *n* = 14; 1 day blast, *n* = 14; 2 days sham, *n* = 7; 2 days blast, *n* = 14; 3 days sham, *n* = 7; 3 days blast, *n* = 14; 7 days sham, *n* = 7; 7 days blast, *n* = 5; 14 days sham, *n* = 7; 14 days blast, *n* = 5; 30 days sham, *n* = 7; 30 days blast, *n* = 7.

Normal sham, lightly anesthetized mice had IOPs ranging from 14 to 15.5 mmHg for the duration of the study (Figure 1). Blast-exposed mice had a baseline IOP of 14 ± 1.6 mmHg (avg ± sd). The IOP increased to 18 ± 1.4, 19 ± 1.4, and 18 ± 2.4 mmHg at 1, 2, and 3 days after blast, respectively. At 7 days post-injury and thereafter the IOP was similar to baseline levels.

### Early, but Not Ongoing, RGC Death After Blast Injury

**FIGURE 2:**
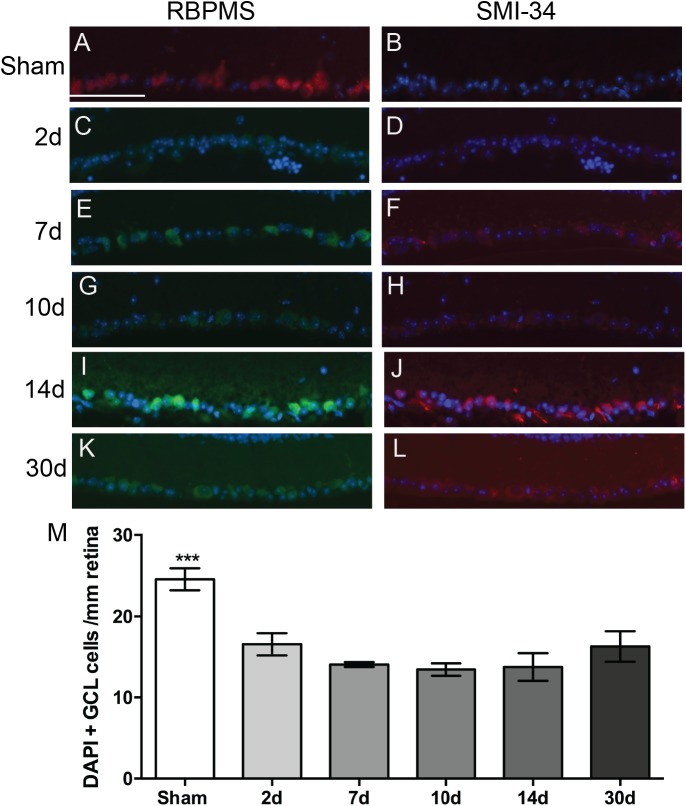
Effect of blast on the RGCs. **(A–L)** Representative fluorescence micrographs of retina cross-sections labeled with anti-RPBMS (red in **A**; green in **C,E,G,I,K**), SMI-34 (red; **B,D,F,H,J,L**) and DAPI (blue) from: **(A,B)** sham, or **(C,D)** 2d, **(E,F)** 7d, **(G,H)** 10d, **(I,J)** 14d, and **(K,L)** 30 d post-blast mice. **(M)** Quantification of nuclei in the GCL over time after injury. Error bars represent s.d. Asterisks indicate that sham was different from all other groups at *p* < 0.001. Scale bar represents 50 μm and is applicable to all images in the figure. *N* = 3 for all groups.

We immunolabeled retinas with anti-RBPMS, a marker for all RGCs, and anti-phosphorylated neurofilament-H, a marker for neuronal degeneration. We also labeled with the nuclear marker, DAPI. In sham mice, there was RPBMS immunolabeling of RGCs (red), but no SMI-34 labeling (red) (Figure 2A,B). At all time-points post-blast, RBPMS immunolabeling could be detected although the brightness of the labeling varied (Figure 2C,E,G,I,K; green). Labeling with SMI-34 was still absent at 2 days (Figure 2D). In contrast, labeling with SMI-34 was detectable at 7, 10, 14, and 30 days post-injury (Figure 2F,H,J,L; red). Phosphorylated neurofilament accumulates in neurons when the axons are degenerative, thus this data suggests that there is axon degeneration at 7 days and later after injury. Since labeling with RBPMS was not consistently present at high levels after injury, we were unable to accurately quantify RGCs using this immunohistochemical approach. Therefore, we instead quantified DAPI-positive cells in the ganglion cell layer (GCL) of the retina, recognizing that displaced amacrine cells, astrocytes, and microglial cells would be included in the total numbers. Using this approach we detected fewer cells in the GCL at all post-blast time-points as compared to shams (Figure 2M). There was no statistically significant difference between post-blast groups.

### Blast-Exposure Does Not Cause Distal Axonopathy

**FIGURE 3:**
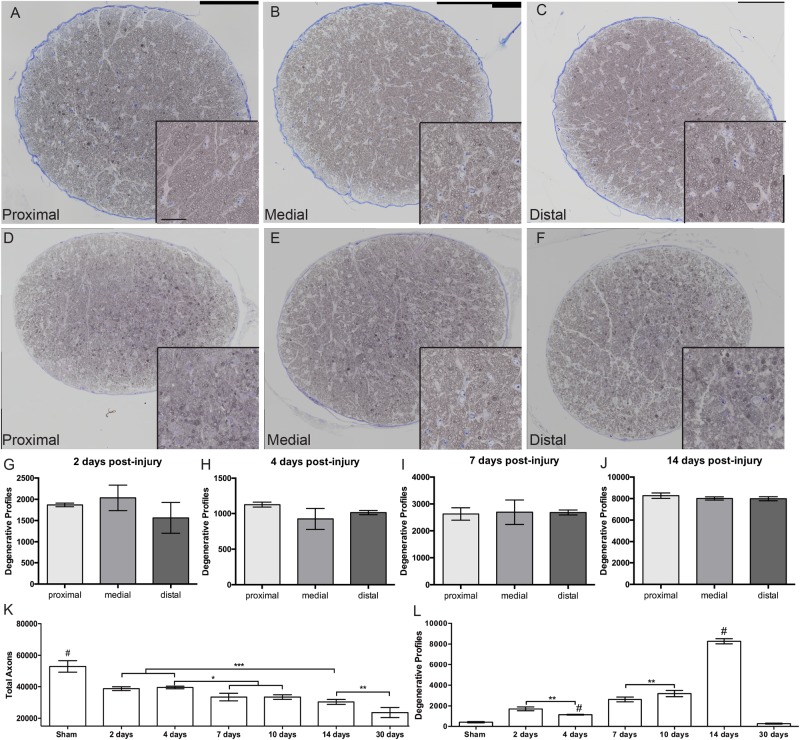
Progression of ON degeneration after blast. **(A–F)** Representative bright-field micrographs of cross-sections from proximal **(A,D)**, medial **(B,E)**, and distal **(C,F)** regions of the ON at 7 days **(A–C)** and 14 days **(D–F)** post-blast. Insets are higher magnification images of the respective ONs. Scale bar for all insets = 20μm. **(G–J)** Quantification of degenerative axon profiles in each spatial location over-time post-injury; **(G)** 2 days, **(H)** 4 days, **(I)** 7 days, and **(J)** 14 days. There was no difference between spatial locations within any time-point. Data shown is mean ± s.d. **(K)** Quantification of total axons over time post-injury. The number of total axons was decreased at 2 days post-injury due to the primary event. Further decreases were detected at 7 and 10 days, 14 days, and 30 days post-blast. **(L)** Quantification of degenerative axon profiles over time post-injury. Degenerative axon profiles from the injury event were still present at 2 days after injury, but was decreased at 4 days. A slight increase was again detected at 7 and 10 days after injury. The peak of degeneration was at 14 days post-injury. Ongoing axon degeneration was completed by 30 days after blast. ^∗^*p* < 0.05, ^∗∗^*p* < 0.01, ^∗∗∗^*p* < 0.001, #*p* < 0.0001. Sham, *n* = 6; 2 days, *n* = 5; 4 days, *n* = 4; 7 days, *n* = 4; 14 days, *n* = 5; 30 days, *n* = 6.

To investigate if blast-induced axon degeneration occurs in a particular region of the ON or progresses in a particular direction over time we assessed cross-sections from proximal, medial, and distal regions of the ON over time (Figure 3). Within each time point the histology looked similar at each location along the nerve (Figure 3A–F). Quantification of degenerative axon profiles confirmed a lack of difference between the proximal, medial, or distal regions of the ON at all time points examined (Figure 3G–J).

Two days after injury, there was a significant increase in degenerative profiles and loss of total axons as compared to shams (Figure 3K,L). At 4 days after injury there were fewer degenerative profiles and the total axon number was unchanged. At 7 and 10 days post-blast there was a slight but statistically significant decrease in total axons and a correlative increase in degenerative profiles. There was a strikingly large increase in degenerative profiles at 14 days after injury (Figure 3L), in agreement with our previous studies (Bernardo-Colon et al., 2018). At 30 days after blast there was a 55% reduction in the total number of axons as compared to sham, but the number of degenerative profiles was no longer significantly different from sham (Figure 3K,L). This suggests that ongoing degeneration had ceased.

### Localization and Timing of Blast-Induced Axon Transport Deficits

**FIGURE 4:**
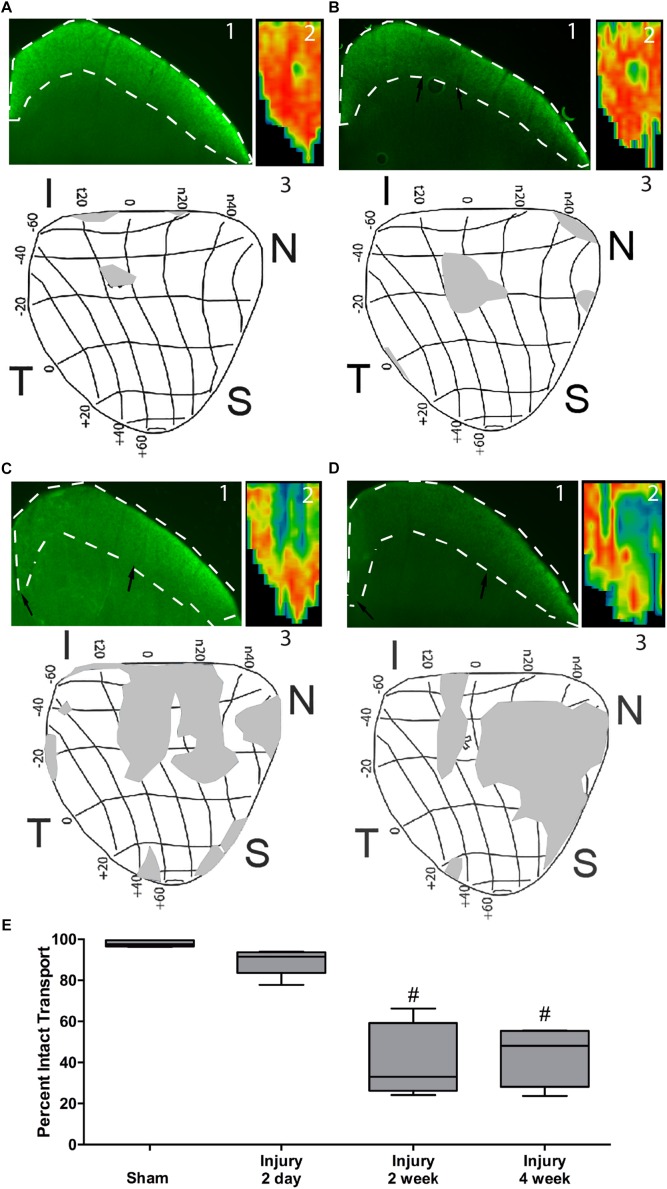
Axon transport deficits after blast injury. A representative fluorescence micrograph of the superior colliculus (1), heat map of total superior colliculus fluorescence (2), and a retinotopic map of transport deficits (3) are shown for sham, *n* = 7, **(A)**, and 2 days, *n* = 6, **(B)**, 14 days, *n* = 5, **(C)**, and 30 days, *n* = 5, **(D)** post-blast animals. Black arrows indicate edges of transport loss in the superior colliculus. Asterisk indicates the location of the ON head in the retinotopic maps. The labels around the maps indicate eccentricity coordinates of the visual field. Abbreviations I, N, S, T indicate inferior, nasal, superior, and temporal quadrants of the retina, respectively. **(E)** Quantification of total axon transport over time post-blast. #*p* < 0.0001 compared to Sham.

In sham animals, the RGC axons actively transport labeled CTB to throughout the superior colliculus as shown by bright fluorescence and red heat map (Figure 4A1,2). The retina representation of transport to the superior colliculus shows complete transport with the exception of the representation of the ON head (Figure 4A3). At 2 days after blast there were small regions with decreased fluorescence indicative of a loss of transport (Figure 4B1,2). These regions were in areas representative of far peripheral retina (Figure 4B3). Transport deficits were similar at 14 and 30 days after blast (Figure 4C,D). There appeared to be a progression of deficits from the periphery to the center of the retina representation of the superior colliculus (Figure 4C,D). Quantification of fluorescence in the superior colliculus showed a trend toward a decrease in transport at 2 days post-blast that did not reach statistical significance (Figure 4E). Transport continued to decline such that at the peak of axon degeneration (14 days after injury) only 41% ± 18 (avg ± sd) axon transport was detected. The percent transport was similar at 30 days after injury. Thus there was a close correlation in timing and level of axon degeneration and transport deficits.

### Astrocytes Are Reactive but Do Not Change Their Overall Pattern or Orientation After Trauma

**FIGURE 5:**
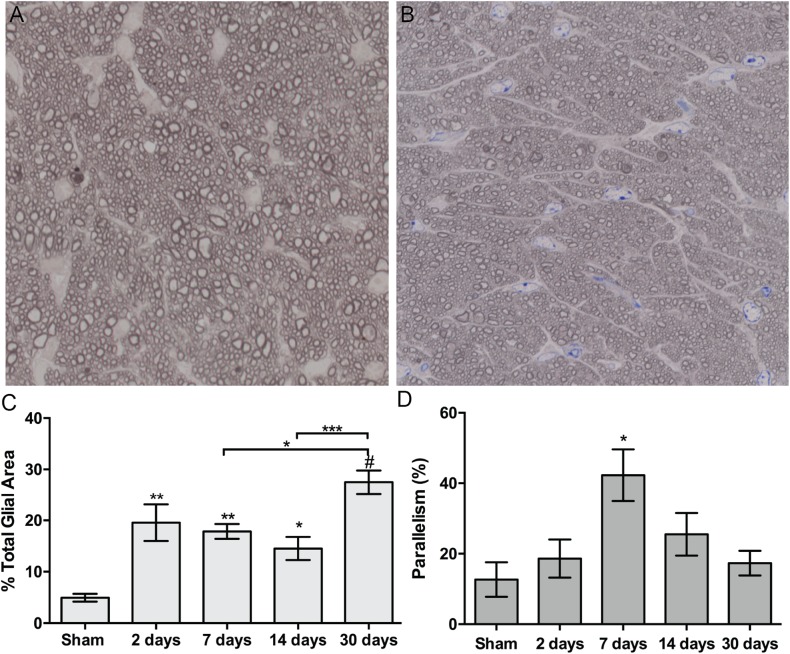
Quantification of ON astrocyte responses to blast injury. **(A,B)** Brightfield micrographs of sham **(A)** and 7 days post-blast **(B)** ON showing glial changes after blast injury. **(C)** Quantification of percent glial area over time after injury. Asterisks above bars indicate differences compared to sham, ^∗^*p* < 0.05, ^∗∗^*p* < 0.01, ^∗∗∗^*p* < 0.001, #*p* < 0.0001. **(D)** Quantification of percent parallelism over time after injury, ^∗^*p* < 0.05. Sham, *n* = 7; 2 days, *n* = 6; 7 days, *n* = 7; 14 days, *n* = 12; 30 days, *n* = 11.

**FIGURE 6:**
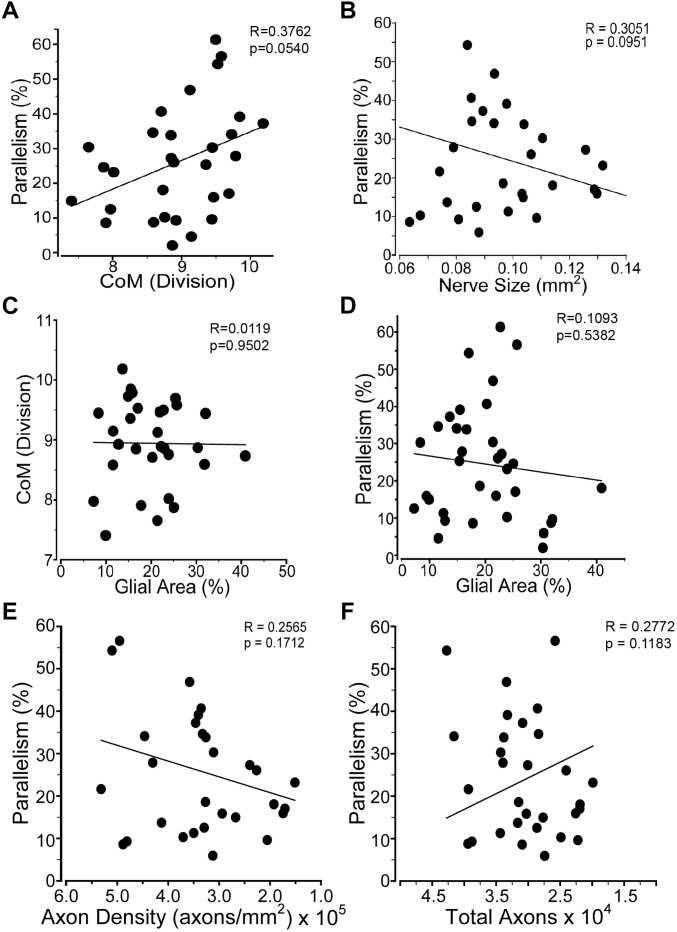
Glial distribution in the ON after injury. Graphs comparing: **(A)** percent glial parallelism and glial center of mass (CoM); **(B)** percent glial parallelism and ON cross-sectional size; **(C)** CoM and percent glial area; **(D)** percent parallelism and percent glial area; **(E)** percent parallelism and axon density; and **(F)** percent parallelism and total axons. Sham, *n* = 7; 2 days, *n* = 6; 7 days, *n* = 7; 14 days, *n* = 12; 30 days, *n* = 11.

Astrocyte size and process distribution appears altered at 7 days post-blast (Figure 5A,B). Astrocyte processes aligned along a more common axis at 7 days after blast, while sham processes appeared to orient at random. We previously published that blast injury causes an increase in glial area in the ON at 2- and 4- weeks after injury, concordant with increased axonal degeneration (Bernardo-Colon et al., 2018). Here we have added additional time points post-blast to better describe injury pathogenesis. We detect an increase in glial area at 2 days after blast as a result of the primary injury (Figure 5C). The percent glial area trended down at 7 and 14 days after injury when compared to the levels at 2 days after injury, but were still higher than shams. Intriguingly, glial coverage increased at 30 days after blast, suggesting a response to the peak of axon degeneration at 14 days. No statistically significant change in glial center of mass was detected at any time after injury (data not shown). The percent of glial parallelism, a measure of process orientation along a common axis, was increased transiently at 7 days after blast (Figure 5D). To further assess the glial response to blast, we compared glial parallelism and center of mass to nerve size, percent glial area, axon density, or total number of axons (Figure 6A–F). No statistically significant changes were detected. Thus, blast injury induces: (1) an increase in glial area following the two peaks of axon degeneration, and (2) a transient increase in glial parallelism in between the two peaks of axon degeneration.

### Blast-Induced Trauma Increases Levels of IL-1 Related Proteins Only

**FIGURE 7:**
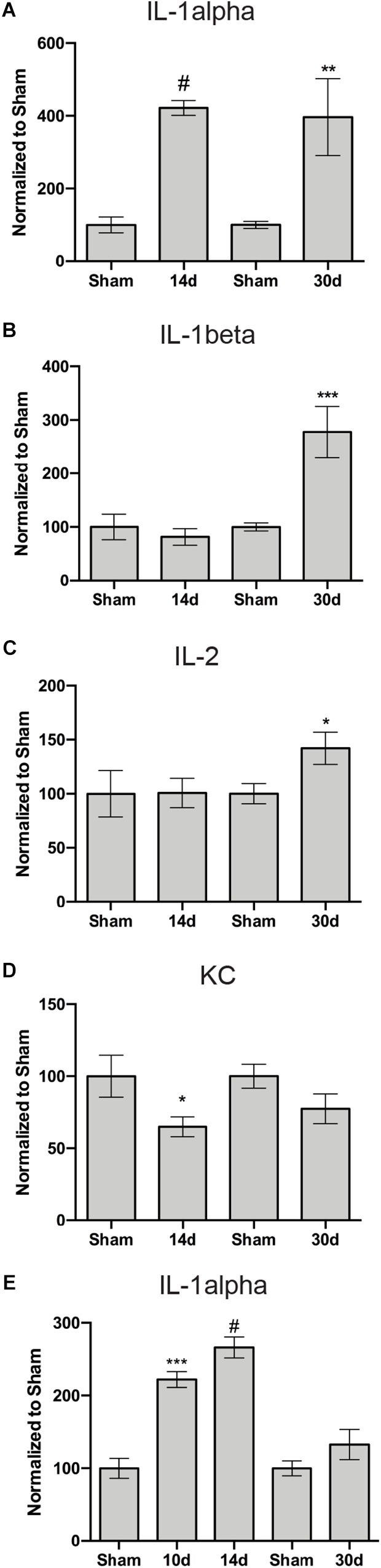
Inflammation-associated proteins with altered levels in the retina **(A–D)** or ON **(E)** after blast. Graphs show: **(A,E)** IL-1α; **(B)** IL-1β; **(C)** IL-2; and **(D)** KC. ^∗^*p* < 0.05, ^∗∗^*p* < 0.01, ^∗∗∗^*p* < 0.001, #*p* < 0.0001. All analytes are presented in Table 1. The n for each is provided in Table 1.

We performed a multiplex cytokine ELISA on retinas and ONs from sham and blast-injured animals. The majority of cytokines and chemokines detected in the ELISA did not change at any time after blast examined (Table 1). In the retina, we detected elevated IL-1α at both 14 and 30 days post-blast (Figure 7A). We also detected elevated IL-1β at 30 days post-blast (Figure 7B), consistent with our previously published results (Bernardo-Colon et al., 2018). In addition we detected an increase in IL-2 at 30 days post-blast (Figure 7C), and a statistically significant decrease in the chemokine KC/CXCL1 at 14 days, but not 30 days after blast (Figure 7D). In the ON, the only cytokine altered after blast was IL-1α, which was statistically significantly elevated at 10 and 14 days after blast, and trended high at 30 days after blast (Figure 7E).

**Table 1:** Quantification of cytokines and chemokines in the retina and ON after injury.

	14d retina	30d retina	10d ON	14d ON	30d ON
G-CSF	ND	89.7±18.0(2)	101.6±8.7(2)	89.8±5.0(3)	75.02±2.2(4)
GM-CSF	108.3±18.3(3)	77.0±39.2(3)	68.4±21.4(4)	98.2±17.8(5)	52.1±18.3(3)
IFNγ	243.4(1)	94.3±17.7(2)	ND	97.8(1)	149.3±76.1(7)
IL-1α	421.6±58.1(8)^∗^	396.5±299.4(8)^∗^	222.0±21.7(4)^∗^	266.1±32.4(5)^∗^	132.5±58.6(8)
IL-1β	81.4±43.7(8)	278.0±134.7(8)^∗^	84.2±10.5(4)	103.1±11.9(5)	91.2±3.5(3)
IL-2	100.7±38.3(8)	142.0±42.3(8)^∗^	82.6±24.9(3)	85.0±13.4(5)	107.9±31.6(8)
IL-4	ND	50.0±36.3(7)	ND	ND	88.1±9.1(4)
IL-5	ND	101.5±0(2)	ND	ND	67.7±14.9(3)
IL-6	ND	64.5±11.2(3)	ND	ND	84.5±24.2(4)
IL-7	ND	98.1±1.8(2)	ND	88.6±5.1(3)	93.6±18.6(4)
IL-9	83.5±28.4(8)	74.1±26.6(4)	77.8±52.9(4)	95.4±15.6(5)	119.4±63.6(8)
IL-10	65.2±49.6(7)	63.6±41.1(4)	68.9±19.4(4)	88.4±11.6(5)	69.3±29.3(6)
IL-12(p40)	ND	ND	ND	79.6±3.7(3)	96.2±32.6(3)
IL-12(p70)	68.4±18.9(5)	73.1±18.2(3)	ND	90.6(1)	107.3±17.6(4)
IL-15	ND	82.4(1)	ND	ND	105.0±55.5(4)
IL-17	ND	104.7±33.4(5)	59.2±25.9(4)	88.1±11.4(5)	97.8±16.4(4)
IP-10	93.8±20.6(6)	103.6±7.6(2)	ND	ND	127.6±130.0(8)
KC	64.9±16.9(6)	77.4±20.7(4)	81.3±56.9(3)	58.9±19.0(5)	92.3±21.0(4)
MCP-1	ND	85.0±23.3(4)	85.1±7.7(3)	94.8±15.9(5)	89.6±30.9(7)
MIP-1α	106.7±27.3(8)	72.0±13.2(4)	79.9±15.1(3)	89.1±17.7(5)	129.7±137.6(8)
MIP-1β	ND	ND	78.1±44.3(4)	111.0±13.1(5)	99.4±4.0(4)
MIP-2	135.1±95.8(8)	75.4±31.6(4)	62.3±3.6(3)	78.8±11.4(4)	64.4±26.1(4)

*^∗^Indicates statistically significant difference, see Figure 6. Values are average±s.d. (n).*

## Discussion

In explosive blasts, propagation of the over-pressure blast wave through the body can cause axon shearing and stress to which long axons appear to be most vulnerable (Garman et al., 2011; Koliatsos et al., 2011). Computer modeling of blast exposure to the eye shows elevated IOP, but these models typically only assess changes that occur during or immediately after blast (Rossi et al., 2012; Bhardwaj et al., 2014; Esposito et al., 2015; Karimi et al., 2016; Notghi et al., 2017). Surprisingly, we detected a 20% elevation in IOP that lasted for 3 days after the last blast exposure. Sustained elevation in IOP is typically associated with glaucoma, a progressive optic neuropathy. In addition to chronic models of elevated IOP, there are acute models that induce a short time course of IOP elevation, similar in duration to what we detected in our trauma model, which also induces ON damage (Valiente-Soriano et al., 2015; Ou et al., 2016). However, the magnitude of the IOP increase is greater in these acute models of ocular hypertension than what we detected after blast. Yet, the amount of axon degeneration detected in our animals was much higher at the same time-point. It is feasible that transient corneal edema that was not visible upon gross exam was responsible for the transient elevation in IOP that we detected. Together this suggests that axon degeneration in our model is not due primarily to post-injury elevated IOP.

We detected a rapid decrease in nuclei present in the GCL at 2 days after blast, without further loss over time. This is comparable to the time course of RGC loss recently reported after ON crush or transection (Sánchez-Migallón et al., 2018).

However, in the absence of a reliably detectable RGC-specific marker after blast injury, it leaves the possibility that some of the cell loss may have been due to death of displaced amacrine cells or astrocytes, or migration of microglial cells out of this layer, potentially into the ON. We also detected an accumulation of phosphorylated neurofilament in the cell bodies of the remaining RGCs that was first evident at 7 days and was greatest at 14 days after injury, suggesting that secondary axon dysfunction begins at 7 days post-injury. The decrease in GCL nuclei at 2 days correlates with the presence of fewer axons at this time-point and suggest that loss of these cells and their axons was due to the initial injury event. It is interesting that no secondary loss of GCL nuclei was detected despite a delayed peak of axon degeneration at 14 days post-injury and loss of axons at 30 days post-injury. This could, in part, be due to infiltration of microglia into the GCL, which would counteract decreases in RGC nuclei since DAPI detects all nuclei in a cell-type independent manner. This should be explored in future studies. Altogether these data demonstrate that RGC loss does not precede axon degeneration. It is feasible that there is additional RGC loss at later time-points, after the loss of axons at 14–30 days. It is interesting to note that the presence of phosphorylated neurofilament in the RGCs at 30 days post-blast suggests that the remaining axons are not entirely healthy. Future studies should explore the long-term effects of repeat blast exposure on the RGCs and axons.

Our model showed comparable axon degeneration at multiple locations along the length of the ON. This suggests that there is not a particular region of the ON that is susceptible to injury from blast wave forces. This is in contrast both to glaucoma, which shows a distal to proximal pattern of axon degeneration progression, and direct TON, which obviously has a specific area of injury in the ON. Further, our data suggests two stages of axon degeneration. The first appears to occur prior to 2 days after blast since there was already a significant loss of total axons and cells in the GCL at this time. This was likely a direct result of the initial injury. The second stage was first detectable at 7—10 days post-injury and peaked at 14 days post-injury and did not correlate with additional loss of cells in the GCL. This stage was likely due to the activity of molecular pathways initiated by the injury event (Bernardo-Colon et al., 2018; Figure 7). The staging of axon degeneration and GCL cell loss we detect here is different from the progressive degeneration that occurs in glaucoma or the rapid, acute degeneration that occurs in models of direct traumatic optic neuropathy (Calkins, 2012).

We detected axon transport deficits that appeared to correlate with axon degeneration, rather than preceding frank axon degeneration or cell death as in models of glaucoma (Crish et al., 2010; Calkins, 2012; Valiente-Soriano et al., 2015). However, we may have missed subtle decreases at intermediate time points or in individual axons. Deficits in axon transport machinery have been detected at the level of the individual axon in blast-induced TBI models by measuring levels of phosphorylated neurofilament in different regions of the neuron (Saljo et al., 2000). Further, the spatial location of axon transport deficits was different between our model and the microbead occlusion model of glaucoma. In the microbead occlusion model, transport deficits were first detected in the inferior nasal quadrant and progressed toward the superior nasal quadrant before then spreading temporally (Lambert et al., 2011). In our model of ITON, axon transport loss after the primary insult is detected centrally and in the far periphery. The secondary degeneration causes transport deficits to increase, moving from the periphery to the center of the retinotopic map. The reason for this is unclear and should be an area of active investigation.

An important role for inflammation has been suggested for both traumatic and glaucomatous optic neuropathies, thus we also explored alterations in the astrocytes and cytokine/chemokine levels. Unlike in the DBA/2J model of glaucoma, we did not detect decreased parallelism or CoM of the ON astrocytes (Cooper et al., 2016, 2018). In fact we detected a trend toward elevated parallelism as CoM increases, which is the opposite to what we have detected in the DBA/2J model of glaucoma. Further, although increases in TNFa, and IL6 have been shown to contribute to glaucoma pathogenesis (Agarwal and Agarwal, 2012; Echevarria et al., 2017), we do not detect increases in either in our model, in fact, TNFa was below the detection limit in our assay. However, we did detect increases in IL-1α and IL-1β, which are also elevated in glaucoma (Adornetto et al., 2019). Increases in IL-1 family proteins is indicative of activation of the inflammasome pathway, which has received increasing attention in both the fields of glaucoma and TBI (Mortezaee et al., 2018; Adornetto et al., 2019). The early increase in IL-1α in both the retina and ON suggests that it is a driver of pathology. Interestingly, a recent paper suggests that IL-1α induces cortical astrocytes to convert to a neurotoxic, reactive, A1, phenotype (Liddelow et al., 2017). Future studies are needed to determine if this is the case in our model. The continued elevation of IL-1α levels at 30 days in the retina, but not the ON may suggest that pathogenesis may continue longer-term in the retina. Further, differences in cytokine levels between ON and retina could also be due to the higher concentration of astrocytes and oligodendrocytes in the ON, while the retina is enriched for neurons. Finally, we recognize that microglia may play an important role in ON pathology due to glaucoma or trauma. We intend to explore any role of these cells in our model of ITON in future studies.

## Conclusion

This study shows that ITON has a neuropathology distinct from direct TON or glaucoma. Finally, the increases in IL-1 related proteins and oxidative stress in our model and models of glaucomatous optic neuropathies suggests that therapies that target these pathways might be effective in both conditions.

## Data Availability

The raw data supporting the conclusions of this manuscript will be made available by the authors, without undue reservation, to any qualified researcher.

## Ethics Statement

All procedures were performed in accordance with the AALAC and the Association for Research in Vision and Ophthalmology guidelines and the VUMC Institutional Animal Care and Use Committee approved protocol.

## Author Contributions

TR conceived and designed the study, supervised implementation, performed the data analysis, and wrote the manuscript. AB-C performed the blast-exposure experiments, axon quantifications and ELISA, and edited the manuscript. VV performed the microscopy, digital quantitative analysis of fluorescence and histology, astrocyte analysis, and edited the manuscript. SN performed ELISAs and edited the manuscript. MC assisted in astrocyte analysis and edited the manuscript. DC supervised the data analysis and edited the manuscript.

## Conflict of Interest Statement

The authors declare that the research was conducted in the absence of any commercial or financial relationships that could be construed as a potential conflict of interest.
